# Advancing DIEP Flap Surgery: Robotic-Assisted Harvest Reduces Pain and Narcotic Use

**DOI:** 10.3390/jcm14155204

**Published:** 2025-07-23

**Authors:** Chloe V. McCreery, Amy Liu, Peter Deptula, Daniel Murariu

**Affiliations:** 1 John A. Burns School of Medicine, University of Hawai’i, Honolulu, HI 96813, USA; chloemcc@hawaii.edu; 2 Department of Surgery, John A. Burns School of Medicine, University of Hawai’I, Queen’s University Tower, 1356 Lusitana Street, 6th Floor, Honolulu, HI 96813, USA; ailiu@hawaii.edu

**Keywords:** robotic deep inferior epigastric perforator flap, autologous breast reconstruction, breast reconstruction, postoperative narcotics use

## Abstract

**Background:** Robotic deep inferior epigastric artery perforator (DIEP) flap surgery is a technique used for autologous breast reconstruction to maintain the integrity of the rectus abdominis muscle while also utilizing robotic assistance for flap harvest. This study assesses postoperative outcomes of patients undergoing robotic DIEP flap reconstruction through the measurement of postoperative pain, narcotics use, and antiemetic usage. **Methods**: A retrospective analysis was performed for patients undergoing robotic DIEP flap breast reconstruction between March 2024 and March 2025. Postoperative pain scores (1–10 scale), narcotics usage (measured in oral morphine equivalents), antiemetic usage, and complications were recorded. Patient outcomes were compared to a control group of 40 patients who had undergone abdominal-based free flap breast reconstruction. **Results**: Overall, 14 patients underwent robotic DIEP flap breast reconstruction, representing 24 breasts. The average patient age was 56.5 (range: 30–73). Ten patients underwent bilateral breast reconstruction, and four underwent unilateral breast reconstruction. The average length of stay postoperatively was 4.86 days (±1.23 days), and the return of bowel function occurred in 1.29 days (±0.47 days). No patients experienced an unplanned return to the OR or flap failure. Average pain scores on postoperative day 1 (POD1), 2 (POD2), and 3 (POD3) were 4.0 (±0.6), 3.4 (±0.6), and 2.93 (±0.5), respectively. Average antiemetic usage totalled 1.25 doses (±0.25). Average daily OME use was 27.7 (±5.0) for POD1, 25.96 (±6.3) for POD2, and 21.23 (±7.11) for POD3. This averaged to a total hospital OME use of 74.9 (±15.7) per patient. Patients undergoing robotic DIEP flap reconstruction required a significantly lower narcotics dosage, as well as a lower antiemetic dosage, during the first three days postoperatively compared to the control abdominal free flap group. Average pain scores in the robotic DIEP flap reconstruction patient group were also significantly decreased, specifically in POD2 and POD3. **Conclusions**: The robotic DIEP flap offers advantages in autologous breast reconstruction compared to other abdominal free flap reconstructive methods. In this limited retrospective study, the use of the robotic DIEP flap lowers chances of flap failure and complications, while also improving narcotics use, antiemetic use, and postoperative pain.

## 1. Introduction

Breast cancer is the most commonly diagnosed cancer among women worldwide. In 2022, 2.3 million women were diagnosed with breast cancer [1]. It is estimated that 12.8% of women will develop breast cancer in their lifetime. Although mortality from breast cancer has been slowly decreasing due to improved diagnostic techniques, the incidence of breast cancer is rising, particularly in women younger than 50 years [2,3]. Treatment typically involves surgical resection, which may remove part or all of the breast, including the nipple–areola complex, as well as lymph nodes or glandular tissue. In addition to surgical resection, adjuvant therapies, such as radiotherapy, immunotherapy, and chemotherapy, may be employed to further prevent tumor growth and the spread of micrometastases that were not removed in the resection, while also reducing mortality and improving the quality of life for the patients after the procedure [4,5]. Adjuvant therapy, when performed with surgical resection, can further decrease breast cancer mortality by up to 21% depending on cancer stage and progression [6].

Surgical resection may also be performed preventatively in patients with high risk of breast cancer. Most commonly, preventative surgical resection is performed for patients with gene mutations in BRCA1, BRCA2, PTEN, and p53 for patients with family histories of breast cancer, and is not typically recommended to persons with an average risk of breast cancer [7]. Inherited gene mutations are found in less than 25% of breast cancer cases, and of these genes, BRCA1 and BRCA2 are the most common cause of hereditary breast cancer [8]. Bilateral mastectomy, particularly in patients with BRCA1 or BRCA2 mutations, has been shown to increase overall survival and reduce breast cancer risk by over 85% [9,10].

It is estimated that over 80,000 mastectomies are performed annually in the United States [11]. The most common types of surgical procedures for breast cancer resection include simple/total mastectomy, partial mastectomy/lumpectomy, modified radical mastectomy, radical mastectomy, nipple-sparing mastectomy, and skin-sparing mastectomy [12]. These procedures vary in their degree of tissue and adjacent structure removal, with the radical mastectomy being the most extensive, involving the removal of the whole breast with overlying skin, the nipple and areola, axillary lymph nodes, and the pectoral muscles. Preventative mastectomy of a healthy breast may also be performed in cases of unilateral breast cancer, also known as a contralateral prophylactic mastectomy [13]. Rates of contralateral breast cancer occurrence are estimated to be 1.9% for women with breast cancer, and rates of contralateral breast cancer incidence have increased in the past twenty years [14,15].

In addition to pain and discomfort, breast cancer and mastectomy can have a major psychosexual impact on patients due to loss of identity and perceived loss of femininity, significantly affecting quality of life. This includes depressive symptoms, poor body image, worsened sexual function, and other signs of psychological distress [16,17]. It is estimated that rates of sexual dysfunction in women with breast cancer may be as high as 60 to 70% [18]. To reduce negative psychosexual effects, mastectomy with breast reconstruction has been shown to have improved psychological outcomes. Improved psychological outcomes were found to be increased particularly in cases of autologous reconstruction and in cases of immediate breast reconstruction, as opposed to implant-based or delayed reconstruction [19]. In addition to providing a natural appearance, autologous breast reconstruction also allows for reinnervation through the use of intercostal nerves, allowing for recovery of sensation to the breast area [20,21].

The deep inferior epigastric artery perforator (DIEP) flap has become ubiquitous in autologous breast reconstruction, in addition to reconstruction of other areas of the body, such as the pelvic, perineal, and lower extremity regions, as well as for head and neck reconstruction. It offers numerous advantages; for most patients, this flap offers ample tissue volume while preserving the rectus abdominis muscle, thereby reducing morbidity [22]. Maintenance of the rectus abdominis muscle also better preserves the patient’s abdominal wall strength, which allows for better function of the abdominal region and significantly lower risks of herniation [23,24]. In addition, DIEP flap reconstruction has been associated with lower rates of fat necrosis and shorter postoperative hospital stays in comparison to breast reconstructions with a transverse rectus abdominis myocutaneous (TRAM) flap [25]. These qualities make the DIEP flap a desirable option for autologous breast reconstruction post-mastectomy.

Robotic-assisted methods for DIEP flap harvest further reduce these risks; transperitoneal pedicle dissection results in a significantly smaller incision of the anterior rectus sheath [26,27]. Across surgical specialties, robotic-assisted surgery has been associated with reduced postoperative pain, decreased opiate requirements, and quicker recovery compared to open techniques [28,29,30,31,32,33]. The use of robotic systems also allows for increased precision and improved visualization of the surgical site, further reducing risks of operative complications. In addition, robotic-assisted surgery leads to improved cosmetic outcomes, as it may eliminate the need for large incisions to access parts of the body [34]. This can improve patient self-esteem and promote positive psychological outcomes [35]. This is especially important with regards to negative impacts of breast cancer diagnosis on body image and psychological state [16,17].

Although there are several reports on the feasibility of a robotic harvest approach, there is still a paucity of comparative data assessing the clinical outcomes in robotic versus traditional approaches. In a bibliometric analysis of breast reconstruction papers written between 2011 and 2021, it was determined that the rate of research publication relating to breast reconstruction post-mastectomy has increased annually, suggesting both an increased interest and need for progress in breast reconstruction techniques [36]. This analysis also showed an increased rate of recommendation for breast reconstruction by physicians to patients with breast cancer. Bibliometric analysis of robotic breast surgery has also shown an upward trend of publications in this field, especially relating to reconstruction [37]. Although research interest in breast reconstruction has increased in recent years, data relating to robotic breast reconstruction, including the use of the DIEP flap, remain extremely limited [38,39].

To address this knowledge, gap, this study aims to compare clinical outcomes, including postoperative pain scores, antiemetic requirements, and narcotic use in oral morphine equivalents between patients undergoing autologous breast reconstruction using a robotic versus open technique. By assessing the results of robotic DIEP flap reconstruction compared to traditional approaches such as the TRAM flap or non-robotic DIEP flap, this study will present early findings of this novel procedure and assess its suitability compared to more common approaches.

## 2. Materials and Methods

### 2.1. Data Collection and Comparison to Controls

A retrospective analysis was performed for fourteen patients undergoing robotic DIEP flap breast reconstruction between March 2024 and March 2025. Patient demographics (age, type of procedure: unilateral or bilateral reconstruction, timing of reconstruction relative to mastectomy, previous cancer diagnosis, side of diagnosis, family history of breast cancer, relevant gene mutations), operative time (total operative time, trochar placement time, dissection times, robotic docking times), flap details (including incision lengths, perforators, pedicle length, flap ischemia time per breast), hospital course, and complications (flap failure, unplanned return to operating room) were evaluated. Inpatient postoperative pain scores were recorded on a 1–10 scale, with 10 being the most severe, on postoperative days 1 to 3. Postoperative antiemetic dosages were recorded, as well as narcotics use in oral morphine equivalents (OMEs). If an antiemetic was required for the patient, either ondansetron, metoclopramide, or prochlorperazine was given. Of the fourteen total patients, two patients required the prescription of patient-controlled anesthesia (PCA) pumps. Individual patient data relating to demographics, operative details, and postoperative recovery can be found in Supplementary Table S1.

Outcomes in this group of patients (the “Robo-DIEP” group) were compared to a previously published control group of 40 patients who underwent abdominal-based free flap breast reconstruction [40,41]. Patients in this group utilized either the TRAM flap (*n* = 2), the muscle-sparing transverse rectus abdominis myocutaneous (MS-TRAM) flap (*n* = 30), or the non-robotically assisted DIEP flap (*n* = 8) in their breast reconstruction procedures. The same postoperative protocols were used between institutions. From this control group, OME use on postoperative days 1 through 3, total OME use, antiemetic use, and postoperative pain scores were recorded for comparison with the Robo-DIEP group.

### 2.2. Statistical Analysis

To account for the difference in sample size between the two groups, statistical analysis was performed using a two-sided Welch’s *t* test, which better accounts for unequal sample size and variance between the groups compared to other statistical tests such as the student’s *t* test. Statistical significance was determined by a *p* value less than 0.05. Statistical analysis was performed using the *tsum.test* function from the BDSA library in R (4.4.3).

## 3. Results

**Figure 1:**
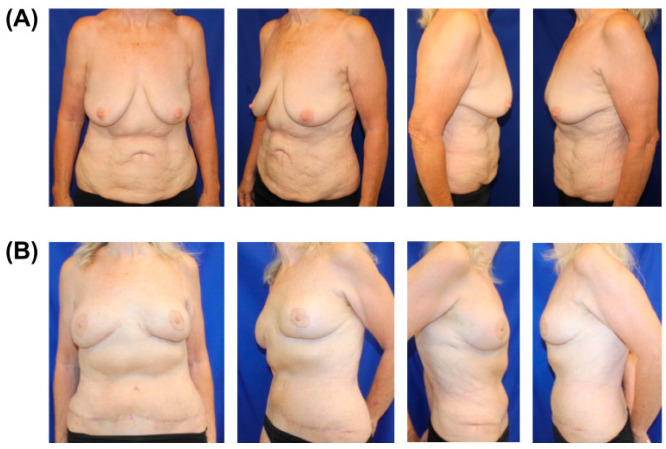
Preoperative (**A**) and postoperative (**B**) images of 65-year-old female with BRCA and previous abdominal liposuction who underwent following stages: first stage—mastopexy; second stage—prophylactic nipple-sparing mastectomies with tissue expander, ADM (acellular dermal matrices), and resensation with nerve grafts; third—removal of tissue expanders and robotic DIEPs; fourth—revisions with removal of skin paddles and upper pole fat grafting. Postoperative photos are 5 weeks post-revision.

Overall, 14 patients underwent robotic DIEP flap breast reconstruction surgery, totalling 24 flaps (the “robo-DIEP” group). The average patient age was 56.5 (range: 30–73). Diagnoses prior to surgery included invasive ductal carcinoma (n = 8, 72.7%), ductal carcinoma in situ (n = 2, 18.2%), and genetic risk from a BRCA1 gene mutation with family history of breast cancer (n = 1, 9.1%) (Figure 1). In this group, ten patients underwent bilateral breast reconstruction, and the remaining four underwent unilateral breast reconstruction. Of the 24 breasts reconstructed, 2 underwent immediate reconstruction after mastectomy (8.3%), 10 underwent reconstruction in a delayed–immediate fashion (41.7%), and 12 underwent delayed reconstruction (50.0%). Patient details between the robo-DIEP compared to the control group are summarized in Table 1. Individual patient details can be found in Supplementary Table S1.

Average pedicle length was 13.98 cm (±1.69 cm, range: 10.5–17 cm). The average fascial incision length was 3.58 cm (±1.58 cm, range: 1.0–6.5 cm). Average number of perforators per flap was 1.67 (±0.7, range: 1–3 perforators), with an average flap ischemia time of 44.67 min (±11.7 min, range: 24–72 min).

The average total operative time for bilateral reconstruction was 12.13 h (±1.8 h, range: 9.80–14.68 h), whereas the total operative time was 7.94 h (±1.5 h, range: 6.45–10.03 h) for unilateral reconstruction. Ther average time for trochar placement was 8.36 min (±3.99 min, range: 4–19 min), with a robotic pedicle dissection time of 31.42 min (±13.9 min, range: 12–59 min) per flap and a peritoneal closure time of 6.3 min (±2.4 min, range: 4–12 min) per flap. The average robotic docking time for the procedure was 32.55 min (±9.26 min, range: 54–142 min) for unilateral reconstruction, and 90.3 min (±31.5 min, range: 26–46 min) for bilateral reconstruction.

Postoperatively, the average length of stay (LOS) was 4.86 days (±1.23 days, range: 3–7 days), with patients experiencing return of bowel function in 1.29 days (±0.47 days, range: 1–2 days). No patients were recorded to have an unplanned return to the OR, or experience flap failure. The average pain scores graded on 1–10 scale on postoperative day 1 (POD1), 2 (POD2), and 3 (POD3) were 4.0 (±0.6), 3.4 (±0.6), and 2.93 (±0.5), respectively. Average total antiemetic usage throughout the hospital stay totalled 1.25 doses (±0.25, range: 0–3 doses). Average daily OME use were 27.7 (±5.0) for POD1, 25.96 (±6.3) for POD2, and 21.23 (±7.11) for POD3. This averaged to a total hospital OME use of 74.9 (±15.7, range: 0–3412.62 OMEs) per patient.

Surgical outcomes of the robo-DIEP group were compared to a control group of patients (n = 40) who had undergone breast reconstruction using other reconstructive methods (DIEP, n = 8; MS-TRAM, n = 30; TRAM, n = 2). Comparing narcotic pain medication usage postoperatively in OMEs, usage in the robo-DIEP group was significantly less for daily usage during POD1 (27.7 ± 5.0 versus 81.9 ± 8.6), POD2 (25.96 ± 6.3 versus 69.3 ± 8.8), and POD3 (21.13 ± 7.11 versus 46.1 ± 5.7). OME use was also significantly lower in the Robo-DIEP group in terms of total usage during their hospital stay, with an average OME use of 74.9 (±15.7) in the Robo-DIEP group and 187.4 (±18.4) in the control group. The robo-DIEP group also used significantly less antiemetic medication, with an average of 1.25 doses (±0.25) compared to the control group, which used an average of 4.5 doses (±0.7).

Average pain scores in the robo-DIEP group were also significantly decreased in POD1, POD2, and POD3 compared to controls. All pharmacologic usage and pain scores are summarized in Table 2, and individual patient postoperative details can be found in Supplementary Table S1.

## 4. Discussion

This study observes the outcomes of 14 patients undergoing robotic DIEP flap breast reconstruction from March 2024 to March 2025. The findings suggest that a minimally invasive approach using robotic-assisted pedicle dissection has benefits in reduction of postoperative narcotic and antiemetic requirements, and enhances recovery through decreased overall pain in the postoperative period in comparison to traditional breast reconstruction approaches.

Since its inception, the DIEP flap has gained popularity as a versatile flap for reconstruction. It is one of the most commonly used flaps for autologous breast reconstruction. However, it is also utilized for the reconstruction of the pelvic and perineal regions after oncologic resection [42,43]. It is also used in distal third lower extremity defects where a paucity of local tissue necessitates free flap reconstruction. And though more infrequently, it is still used for head and neck reconstruction, among other sites [44,45]. The advantages of the DIEP flap, compared to musculocutaneous flaps (such as TRAM), include decreased abdominal donor site morbidity, decreased hospital length of stay, and lower rates of fat necrosis when used in breast reconstruction [25].

Although the preservation of the rectus muscle decreases donor site morbidity, traditional DIEP harvest necessitates incision of the anterior rectus sheath and there is still risk of bulge or hernia [26]. Robotic DIEP was first described in 2020 and utilizes an intra-abdominal approach for dissection of the deep inferior epigastric perforator pedicle, which causes less disruption of the abdominal muscle and fascia [46]. The authors suggest that this novel minimally invasive approach could improve recovery time and reduce postoperative pain [46]. Our study substantiates these findings; patients in the robo-DIEP group had not only reduced OME usage in the first three days of recovery, but also reported significantly less pain during the first three days of recovery.

It is notable that robotic DIEP flap reconstruction requires less opioid use for postoperative pain compared to other reconstructive methods. Opioid use after surgical procedures has been shown in multiple contexts to increase mortality risks and increase the likelihood of subsequent health events and ER visits, even in minor elective surgical procedures [47,48,49]. Use, even at prescribed dosages, is associated with risk dependence and tolerance [50]. Moderate to high levels of opioid use prior to surgical procedures are also correlated with increased mortality risks [48]. The concept of an enhanced recovery after surgery (ERAS) pathway was first introduced in 1997 and has since gained popularity across surgical specialities [51,52]. It was developed to reduce the economic burden on the healthcare system by optimizing postoperative care using a multidisciplinary three-pronged approach to decrease hospital LOS, reduce complications, and promote recovery; preoperative, intraoperative, and postoperative factors contributing to delayed recovery are targeted in ERAS pathways. Pain is one of the principal targets of postoperative recovery optimization in all ERAS protocols [53]. ERAS protocols have also been implemented in recovery after microsurgical breast reconstruction, and the subsequent reduction of postoperative pain and decreased opiate use has been shown to decrease LOS [54]. Emerging technology is usually associated with increased cost; criticism of robotic-assisted procedures often hangs on economic concerns [55,56]. However, higher initial surgical costs may be negated by decreased overall costs associated with opioid use and LOS. Further cost-analysis may be warranted to quantify these differences.

Our findings that pain and narcotic use are significantly lower with the robotic-assisted technique are consistent with prior studies in the literature. Tsai et al. also reported their series of robot-assisted DIEP flaps, and found an improved pain profile amongst their robot-assisted DIEP flap group compared to the conventional DIEP flap group. However, the differences in pain scores on POD1-3 were not statistically significant in their findings [57]. Lee et al. also found improved pain profiles, but with a totally extraperitoneal approach (TEP) to robot-assisted pedicle harvest. The authors found statistically significant improvement in pain scores between 6–24 h with robotic (TEP) harvest compared to their conventional control. The authors similarly noted less narcotic use with significantly reduced doses of fentanyl, which is provided by PCA based on their postoperative protocol [26]. Other minimally invasive means of autologous breast reconstruction are being introduced with similar benefits. Deptula et al. demonstrate that the omental fat augmented free flap, involving the laparoscopic harvest of omental tissues similarly reduces post operative pain, narcotic requirement and antiemetic use [40].

Average rates of flap failure in patients undergoing traditional DIEP flap reconstruction range between 1–2% [58,59]. Aside from partial or total flap failure, the most common complications associated with the DIEP flap procedure include wound complications, hematoma, venous congestion, partial flap loss, and arterial thrombosis [60]. However, in the cases observed in this study, no patients were recorded to experience an unplanned return to the OR, or flap failure. This finding is notable and will be investigated in future studies, with a larger cohort of patients to confirm the reduced rates of flap failure and complications.

A major limitation of this study is the small sample size of the robo-DIEP patient group, in comparison to the control group with 14 breasts and 40 breasts, respectively. Furthermore, there were fewer patients who underwent immediate reconstruction in the robo-DIEP group compared to the control group. The effect of timing on these clinical outcomes is unclear.

The early results and sample size discrepancies between the two groups limit the generalizability and statistical power of the findings. However, given this technique’s recent invention and usage, the authors find that the results of this study provide significant early findings on the effectiveness of robotic DIEP flap reconstruction that will encourage further investigation and improvement of this procedure. Continued investigation with a prospective study and a larger patient cohort will further confirm this study’s findings and limit biases that are inherently present in a retrospective study. This study also included myocutaneous flaps in the control group, and a dedicated study comparing the outcomes in robotic versus conventional pedicle dissection on DIEP flaps alone may better elucidate the benefits of robotic approach. Other notable areas that require further investigation include long-term surgical outcomes (both in terms of complications and patient satisfaction), outpatient recovery and narcotic usage after reconstruction, and long-term recovery and function of the DIEP flap donor site.

## 5. Conclusions

This study showed, through a small patient group, the benefits of robotic DIEP flap breast reconstruction over traditional DIEP flap reconstruction and other reconstructive methods. Patients undergoing the robotic DIEP flap procedure had significantly lower narcotics use and antiemetic use following the surgery, and also reported significantly lower pain scores during the first three days of recovery. In addition, patients in this study who underwent robotic DIEP flap breast reconstruction did not experience an unplanned return to the OR or flap failure. Further usage of this technique on a larger patient population will allow for the better assessment of the robotic DIEP flap’s benefits.

## Figures and Tables

**Table 1:** Patient procedure demographics and procedure details for autologous breast reconstruction in robotic deep inferior epigastric artery perforator (DIEP) flap group versus controls. A “-“ in the table represents zero patients.

	Robo-DIEP (n = 14)	Controls (n = 40)
Procedure Details		
Bilateral Reconstruction	10	23
Unilateral Reconstruction	4	17
Procedure Timing		
Immediate	2	18
Delayed–Immediate	10	0
Delayed	12	22
Reconstruction Method		
Robotic DIEP	14	-
DIEP	-	8
MS-TRAM	-	30
TRAM	-	2

**Table 2:** Postoperative narcotic use, antiemetic use, and average pain scores postoperative day (POD) 1 through POD3 after autologous breast reconstruction. Pain scores were measured using 1 to 10 scale. Postoperative narcotic usage is displayed in oral morphine equivalents. *p* value is calculated via Welch’s *t*-test; *p* < 0.05 is considered statistically significant.

	Robo-DIEP (n = 14)	Controls (n = 40) ‡	*p* Value
Postoperative Pharmacologic Agent Usage			
OME, POD1	27.7 (±5.0)	81.9 (±8.6)	<0.0001
OME, POD2	25.96 (±6.3)	69.3 (±8.8)	<0.0001
OME, POD3	21.13 (±7.11)	46.1 (±5.7)	<0.0001
OME, Total	74.9 (±15.7)	187.4 (±18.4)	<0.0001
Antiemetic, Total	1.25 doses (±0.25)	4.5 doses (±0.7)	<0.0001
Average Pain Scores			
POD1	4.0 (±0.6)	4.8 (±0.4)	0.0002
POD2	3.4 (±0.6)	5.4 (±0.3)	<0.0001
POD3	2.93 (±0.5)	5.0 (±0.3)	<0.0001

‡ Control group consisted of patients undergoing abdominal-based free flap autologous breast reconstruction from previously published studies [27].

## Data Availability

The original contributions presented in this study are included in the article/Supplementary Materials. Further inquiries can be directed to the corresponding authors.

## Supplementary Materials

The following supporting information can be downloaded at: https://www.mdpi.com/article/10.3390/jcm14155204/s1, Table S1: Individual data from the robo-DIEP group patients, related to (A) relevant patient history, (B) operative details, and (C) post-operative recovery.
