# Supplementary material for: Advancing DIEP Flap Surgery: Robotic-Assisted Harvest Reduces Pain and Narcotic Use
Source: J Clin Med. 2025 Jul 23;14(15):5204. doi: 10.3390/jcm14155204 (PMC12346951; doi:10.3390/jcm14155204)
Supplement: Supplementary file 1 [file jcm-14-05204-s001.zip › jcm-3717553-supplementary.pdf]

Table S1 Individual data from the robo-DIEP group patients, related to (A) relevant patient history, (B) operative details, and (C) post-operative recovery.

A) Patient History

Patient Code	Age	Procedure Type	Timing	Diagnosis	Side of Diagnosis	Family History of Breast Cancer	Gene Mutation
1	63	Bilateral	Delayed	IDC	L	No	No
2	69	Bilateral	Delayed	IDC	R	No	No
3	47	Bilateral	Delayed-Immediate	IDC	L	No	No
4	55	Bilateral	Delayed-Immediate	IDC	L	No	No
5	56	Bilateral	Delayed-Immediate	DCIS	R	No	No
6	48	Unilateral	Immediate	DCIS	R	No	No
7	44	Bilateral	Delayed	IDC	L	No	No
8	65	Bilateral	Delayed-Immediate	N/A	N/A	Yes	No
9	73	Unilateral	Immediate	IDC	L	No	No
10	66	Bilateral	Delayed-Immediate	IDC	L	No	No
11	30	Bilateral	Delayed	IDC	L	No	BRCA
12	69	Unilateral	Delayed	DCIS	R	No	No

13	40	Bilateral	Delayed	IDC	R	No	No
14	66	Unilateral	Delayed	IDC	L	No	No

B) Operative Details

Patient Code	Total Operative Time (Hours)	Trochar Placement Time (min)	Pedicle Time, Breast 1 (min)	Pedicle Time, Breast 2 (min)	Closure Time, Breast 1 (min)	Closure Time, Breast 2 (min)	Dock Time (hours)	Right Flap Vessel Length (A/V) (mm)	Left Flap Vessel Length (A/V) (mm)	Pedicle length (cm)	Fascial Incision Length: Breast 1, Breast 2 (cm)	Number of Perforators: Breast 1 ,Breast 2	Ischemia Time: Breast 1, Breast 2 (min)
1	14.68	19	38	59	8	10	2.37	2.7/2.5	3.0/3.5	13,17	6.5,3.5	3,3	39,56
2	13.83	10	22	16	6	5	1.17	2.5/4.0	2.5/>4.0	14,10.5	4.5,5.0	2,2	59,72
3	14.65	5	20	37	5	10	1.55	3.0/>4.0	3.0/>4.0	15,13	4,5	2,2	34,37
4	12.53	10	49	52	5	7	2.3	2.5/>4.0	2.8/>4.0	15,16	3,2	2,1	21,35
5	12.67	11	12	19	5	10	0.9				5,6.5	1,3	51,51
6	10.03	8	59	N/A	3	N/A	0.47		2.7/3.5	13.5	5	2	58

7	10.9	4	23	22	7	5	1.07	2.5/2.5	2.4/3.0	15,14	3,5.8	2,1	39,62
8	11.35	8	30	26	5	6	1.25	3.0/4.0	3.0/>4.0	15,15	2.5,3	2,1	40,30
9	7.48	12	19	N/A	6	N/A	0.5	3.0/4.0		15	2.5	1	45
10	10.58	4	25	27	3	4	1.17	2.5/>4.0	2.8/>4.0	13,14	4.8,1.8	2,1	35,41
11	9.8	5	30	34	6	4	1.3	2.5/>4.0	2.5/>4.0	11,15	2.4,1.5	1,1	40,53
12	7.8	7	32	N/A	8	N/A	0.77		2.5/>4.0	15	3	2	40
13	10.28	8	47	43	4	12	1.97	2.8/>4.0	2.8/>4.0	15,14.5	1,2	1,1	38,41
14	6.45	6	13	N/A	8	N/A	0.43	2.5/3.5-4		11	2.5	1	55

C) Postoperative Recovery

Patient Code	Length of Stay (days)	Pain Score Day 1	Pain Score Day 2	Pain Score Day 3	Patient-Controlled Anesthesia (PCA)?	Number of Days for Return of Bowel Function	Morphine equivalents Day 1	Morphine equivalents Day 2	Morphine equivalents day 3	Total Morphine equivalents	Total Anti-emetic Doses	Total Zofran Doses	Total Reglan Doses	Total Compazine Doses
1	7	7.75	7	4.7	No	1	21.6	27	10.8	59.4	1	1	0	0

2	4	4.125	2.15	0	No	1	22.5	15	5	42.5	1	1	0	0
3	5	4.1	5.25	5.21	Yes	2	744.12	1006.5	1662	3412.62	1	1	0	0
4	4	3.09	0.33	3.4	No	1	48.75	0	0	48.75	3	3	0	0
5	7	5.6	5.22	5.2	No	1	45	60	30	135	1	1	0	0
6	6	2.4	2	2.7	No	2	41.1	30	37.5	108.6	2	2	0	0
7	4	2.56	1.6	2.2	No	1	10	15	10	35	1	1	0	0
8	5	3.8	3.77	3.63	No	1	45	12	4	61	1	1	0	0
9	4	3	3.25	3.55	no	1	50	70	75	195	2	1	1	0
10	6	3.9	3	1.86	No	1	10.8	15	7.5	33.3	1	1	0	0
11	4	7.8	6.3	5.25	Yes	1	540	1482	900	2922	3	1	0	2
12	5	5.2	4.8	3.1	No	1	15	30	15	60	2	1	0	1
13	4	6.6	6.2	4.5	No	2	23	37.5	60	120.5	0	0	0	0
14	3	0.5	1.28	0.33	No	2	0	0	0	0	0	0	0	0
